# Supplementary material for: Vocal cues to eyewitness accuracy are detected by listeners with and without language comprehension
Source: Commun Psychol. 2025 Apr 17;3:65. doi: 10.1038/s44271-025-00237-2 (PMC12006449; doi:10.1038/s44271-025-00237-2)
Supplement: Supplementary file 2 — Supplementary information [file 44271_2025_237_MOESM2_ESM.pdf]

Supplemental material

Gustafsson, Laukka, Elfenbein & Thingujam 2025

S Figure 1a-d. Violin plots showing the distribution of data, with mean and 95% confidence intervals.

S Figure 1a. Accuracy, Free recall

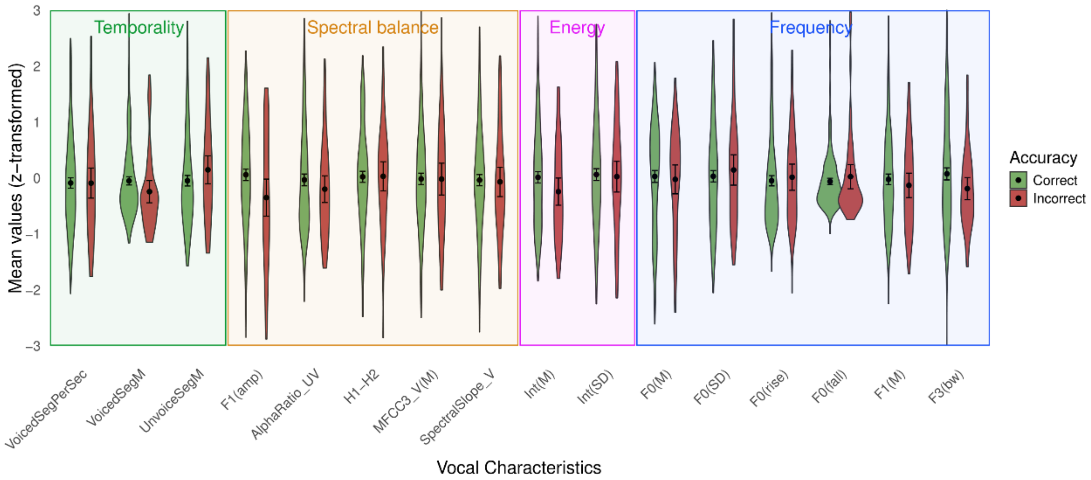

S Figure 1b. Confidence, Free recall

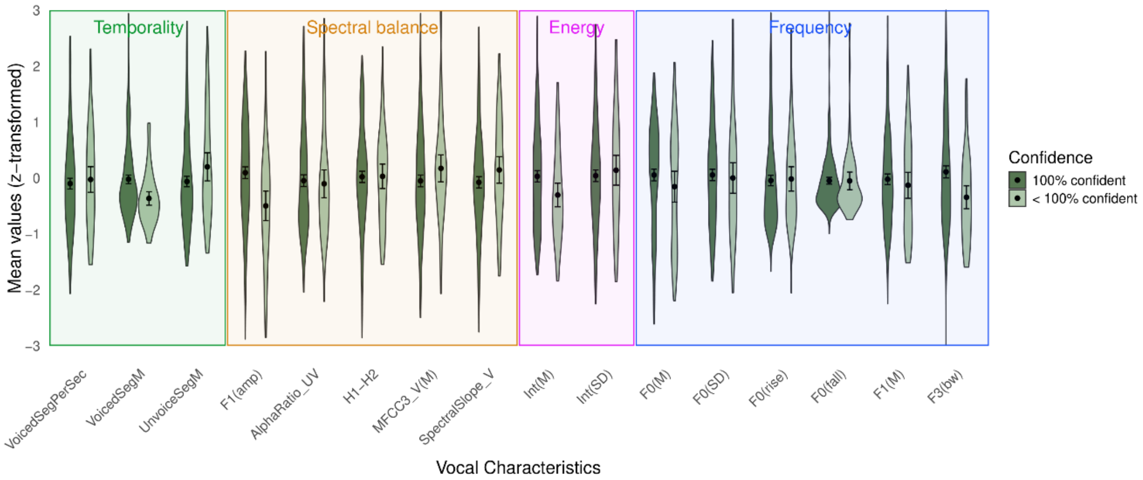

S Figure 1c. Accuracy, Cued recall

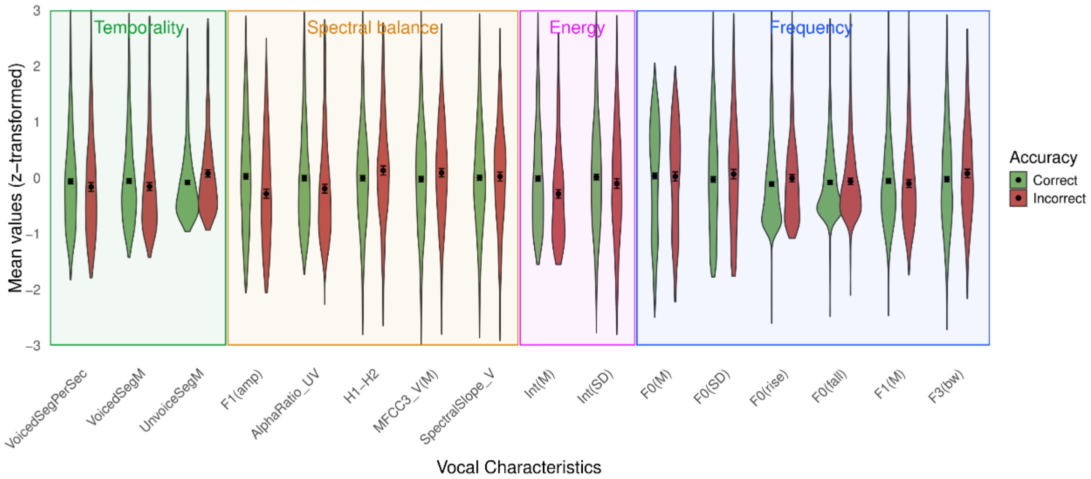

S Figure 1d. Confidence, Cued recall

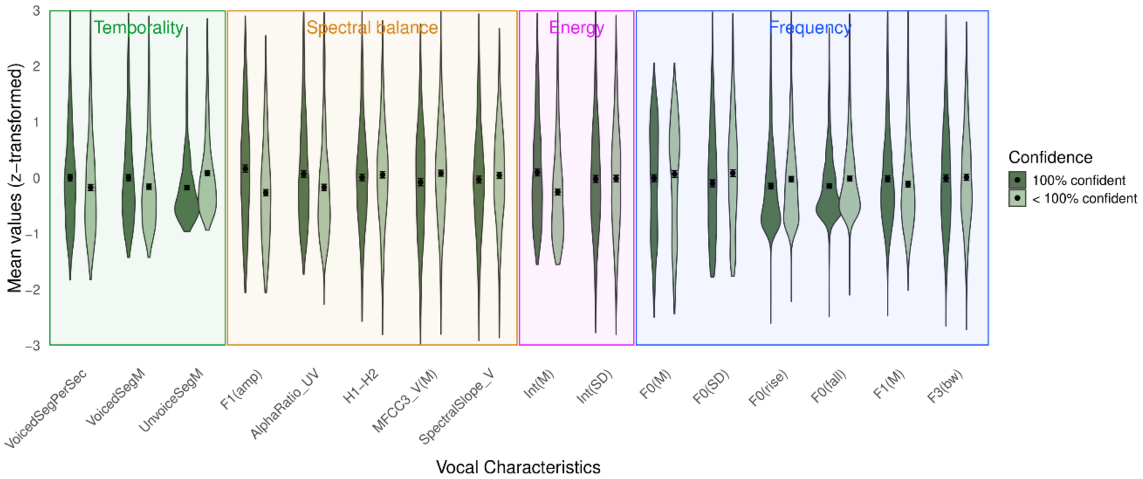

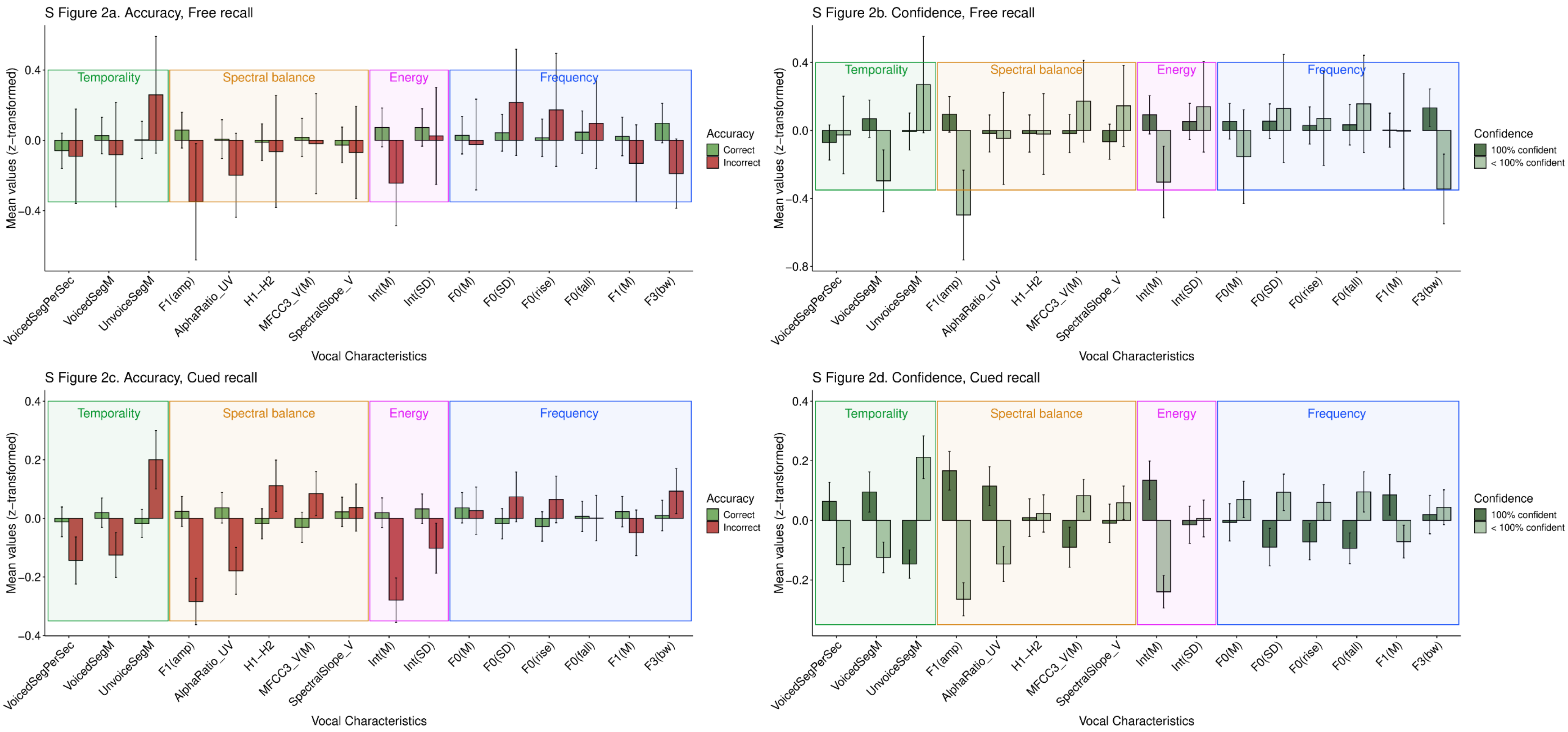

S Figure 2a-d. A zoom in on the means and 95% CIs, without showing the distribution, for a clearer display of the patterns of acoustic parameters across conditions.
