# Supplementary material for: Vocal cues to eyewitness accuracy are detected by listeners with and without language comprehension
Source: Commun Psychol. 2025 Apr 17;3:65. doi: 10.1038/s44271-025-00237-2 (PMC12006449; doi:10.1038/s44271-025-00237-2)
Supplement: Supplementary file 3 — Reporting Summary [file 44271_2025_237_MOESM3_ESM.pdf]

nature portfolio

Corresponding author(s): DBPR Manuscript ID PSCI-23-0856

Last updated by author(s): Mar 15, 2025



# Reporting Summary

Nature Portfolio wishes to improve the reproducibility of the work that we publish. This form provides structure for consistency and transparency in reporting. For further information on Nature Portfolio policies, see our Editorial Policies and the Editorial Policy Checklist.

## Statistics

For all statistical analyses, confirm that the following items are present in the figure legend, table legend, main text, or Methods section.

| n/a | Confirmed | |
|---|---|---|
| ☐ | ☒ | The exact sample size (*n*) for each experimental group/condition, given as a discrete number and unit of measurement |
| ☐ | ☒ | A statement on whether measurements were taken from distinct samples or whether the same sample was measured repeatedly |
| ☐ | ☒ | The statistical test(s) used AND whether they are one- or two-sided<br>*Only common tests should be described solely by name; describe more complex techniques in the Methods section.* |
| ☐ | ☒ | A description of all covariates tested |
| ☐ | ☒ | A description of any assumptions or corrections, such as tests of normality and adjustment for multiple comparisons |
| ☐ | ☒ | A full description of the statistical parameters including central tendency (e.g. means) or other basic estimates (e.g. regression coefficient) AND variation (e.g. standard deviation) or associated estimates of uncertainty (e.g. confidence intervals) |
| ☐ | ☒ | For null hypothesis testing, the test statistic (e.g. *F*, *t*, *r*) with confidence intervals, effect sizes, degrees of freedom and *P* value noted<br>*Give P values as exact values whenever suitable.* |
| ☒ | ☐ | For Bayesian analysis, information on the choice of priors and Markov chain Monte Carlo settings |
| ☐ | ☒ | For hierarchical and complex designs, identification of the appropriate level for tests and full reporting of outcomes |
| ☐ | ☒ | Estimates of effect sizes (e.g. Cohen's *d*, Pearson's *r*), indicating how they were calculated |

*Our web collection on statistics for biologists contains articles on many of the points above.*

## Software and code

Policy information about availability of computer code

| | |
|---|---|
| Data collection | The data and code for Study 1 are publicly accessible at https://osf.io/x7u5g. The data, code and materials for Study 2 are publicly accessible at https://osf.io/x7u5g. |
| Data analysis | The data and code for Study 1 are publicly accessible at https://osf.io/x7u5g. The data, code and materials for Study 2 are publicly accessible at https://osf.io/x7u5g. |

For manuscripts utilizing custom algorithms or software that are central to the research but not yet described in published literature, software must be made available to editors and reviewers. We strongly encourage code deposition in a community repository (e.g. GitHub). See the Nature Portfolio guidelines for submitting code & software for further information.

## Data

Policy information about availability of data

All manuscripts must include a data availability statement. This statement should provide the following information, where applicable:

- Accession codes, unique identifiers, or web links for publicly available datasets
- A description of any restrictions on data availability
- For clinical datasets or third party data, please ensure that the statement adheres to our policy

Data is available on the Open Science Framework (Study 1, https://osf.io/x7u5g; Study 2, https://osf.io/x7u5g). Data for Study 1 contain values for all vocal cues

included in the analyses for all included witness statements. Data for Study 2 contain anonymized data from all participants in the human judgment study. The conditions of ethics approval and consent procedures do not permit the public archiving of the audio files containing the witness statements.

## Research involving human participants, their data, or biological material

Policy information about studies with human participants or human data. See also policy information about sex, gender (identity/presentation), and sexual orientation and race, ethnicity and racism.

| | |
|---|---|
| Reporting on sex and gender | Participants<br>Two-hundred and seventy-seven participants took part in this study (Mage = 24.27, SD = 11.38; 153 women, 120 men, 1 non-binary, 3 non-responses). Participants were recruited from three different countries, Sweden (n = 61, Mage = 37.02, SD = 11.78; 40 women, 17 men, 1 non-binary, 3 non-responses), USA (n = 156, Mage = 19.40, SD = 3.20; 83 women, 73 men) and India (n = 60, Mage = 19.40, SD = 0.91; 30 women, 30 men). |
| Reporting on race, ethnicity, or other socially relevant groupings | All participants gave written consent to participate. Participants from Sweden were recruited from an online recruitment pool (www.accindi.se) and received a movie voucher as compensation; participants from USA were recruited at the Washington University in St. Louis and took part in the study for course credit, and participants from India were recruited from Tripura University and received a monetary compensation of 500 Indian rupees (around 5.6 euro). In the Indian sample, the participants were Bengali (27), Assamese (9), Indian Nepali (1), and tribes of Tripura (18), Manipuri (3), and from other north Indian groups (2). In the American sample, there were 53 Asian or Pacific Islanders, 13 Black or African Americans, 9 Hispanic or Latinos, 75 White or Caucasians, 2 Biracial or Multiracial, and 4 who rated “a race/ethnicity not listed”. All participants in the American and Indian sample reported that they had no Swedish language comprehension skills. |
| Population characteristics | See above |
| Recruitment | See above |
| Ethics oversight | The study was approved by the Swedish Ethical Review Authority (#2018/2030-31/5), as well as by the Institutional Review Board at Washington University in St. Louis. Ethical vetting was not performed for the Indian data collection as it was not required according to the guidelines for psychological research at Tripura University. |

Note that full information on the approval of the study protocol must also be provided in the manuscript.

# Field-specific reporting

Please select the one below that is the best fit for your research. If you are not sure, read the appropriate sections before making your selection.

☐ Life sciences ☒ Behavioural & social sciences ☐ Ecological, evolutionary & environmental sciences

For a reference copy of the document with all sections, see nature.com/documents/nr-reporting-summary-flat.pdf

# Behavioural & social sciences study design

All studies must disclose on these points even when the disclosure is negative.

| | |
|---|---|
| Study description | Quantitative. |
| Research sample | See section above on "research involving human participants, their data, or biological material" |
| Sampling strategy | An a priori power analysis for one-way fixed effects analysis of variance (ANOVA) with 3 groups suggested n = 159, given 80% power to detect a medium effect size (f = .25) at standard 0.05 alpha error probability. We chose a medium effect size in order to focus of the most robust effects while still having enough power also to detect effects that could be smaller yet still have practical and theoretical significance. Please note that this calculation deviates from the preregistration, wherein the medium effect size was erroneously entered as f = .39. We note that our final sample was larger than planned because a greater number of participants than anticipated signed up in the USA. Due to guidelines regarding course credit, all interested participants were welcomed into the study, and we did not exclude otherwise eligible data from analysis. |
| Data collection | The survey was created using Qualtrics and participants completed it online by accessing a link provided by the researcher. Participants were first informed that the purpose of the study was to examine judgments of eyewitness accuracy. They were also informed that the witnesses had watched a staged crime film and gave sincere testimony about what they had seen, and that the task was not about detecting deception, but instead to detect when a statement was correct or incorrect. Furthermore, all participants were informed that they should focus on the “nonverbal aspects of the speech, such as the tone of voice” rather than on the verbal content when making their judgments. The American and Indian participants were additionally informed that the statements were spoken in Swedish and they were not expected to understand the content of the speech. Next, participants went through an audio check to make sure they could hear the audio files. They were then instructed that they would listen to statement excerpts from testimony one-by-one, and that their task was to 1) judge accuracy (correct/incorrect) and 2) judge perceived speaker confidence (Likert scale ranging from 1 “Not confident at all” to 9 “Very confident”). After judging their pool of 100 statements, participants provided demographic information and were thanked for participation. |

| | |
|---|---|
| Timing | Spring 2022 (March 1st to May 1st) |
| Data exclusions | For the pre-used data in Study 1: The current dataset contains data from 51 participants (Mage = 29.45, SDage = 8.23, 66.03% women) because three participants did not consent to further use of their data and data from two participants contained excessive background noise that prevented meaningful audio from being extracted (see more about statement exclusion below). The resulting dataset contained 3,344 statements (76.61% accurate; Mduration = 4.26 seconds, SD = 3.13 seconds).<br><br>For study 2: Stimuli consisted of a selection of the audio clips from Study 1. Specifically, we randomly sampled 5 correct and 5 incorrect responses to cued recall questions from each of the witness testimony statements used in Study 1 (originally from Gustafsson et al., 2022). Out the 51 testimonies, one contained fewer than 5 incorrect statements (n = 4), and was therefore excluded from the sample, leaving us with a pool of 500 witness statements (50% correct).<br>No data was excluded from participants in study 2. |
| Non-participation | No known dropouts. |
| Randomization | Stimuli consisted of a selection of the audio clips from Study 1. Specifically, we randomly sampled 5 correct and 5 incorrect responses to cued recall questions from each of the witness testimony statements used in Study 1 (originally from Gustafsson et al., 2022). Out the 51 testimonies, one contained fewer than 5 incorrect statements (n = 4), and was therefore excluded from the sample, leaving us with a pool of 500 witness statements (50% correct). To prevent fatigue for participants, we split these samples into five sets, grouping the 500 statements into five groups of 100 statements that each contained all sampled statements from 10 witnesses in each group. Participants were then randomly assigned into one of five surveys containing one of these pools of 100 statements. The presentation order of the statements was randomized. All statements were spoken in Swedish and lasted 0.36 – 21.45 seconds (M = 4.44, SD = 3.34). |

# Reporting for specific materials, systems and methods

We require information from authors about some types of materials, experimental systems and methods used in many studies. Here, indicate whether each material, system or method listed is relevant to your study. If you are not sure if a list item applies to your research, read the appropriate section before selecting a response.

## Materials & experimental systems

| n/a | Involved in the study |
|---|---|
| ☒ | ☐ Antibodies |
| ☒ | ☐ Eukaryotic cell lines |
| ☒ | ☐ Palaeontology and archaeology |
| ☒ | ☐ Animals and other organisms |
| ☒ | ☐ Clinical data |
| ☒ | ☐ Dual use research of concern |
| ☒ | ☐ Plants |

## Methods

| n/a | Involved in the study |
|---|---|
| ☒ | ☐ ChIP-seq |
| ☒ | ☐ Flow cytometry |
| ☒ | ☐ MRI-based neuroimaging |

## Plants

| | |
|---|---|
| Seed stocks | *Report on the source of all seed stocks or other plant material used. If applicable, state the seed stock centre and catalogue number. If plant specimens were collected from the field, describe the collection location, date and sampling procedures.* |
| Novel plant genotypes | *Describe the methods by which all novel plant genotypes were produced. This includes those generated by transgenic approaches, gene editing, chemical/radiation-based mutagenesis and hybridization. For transgenic lines, describe the transformation method, the number of independent lines analyzed and the generation upon which experiments were performed. For gene-edited lines, describe the editor used, the endogenous sequence targeted for editing, the targeting guide RNA sequence (if applicable) and how the editor was applied.* |
| Authentication | *Describe any authentication procedures for each seed stock used or novel genotype generated. Describe any experiments used to assess the effect of a mutation and, where applicable, how potential secondary effects (e.g. second site T-DNA insertions, mosiacism, off-target gene editing) were examined.* |
