# Supplementary material for: Vocal cues to eyewitness accuracy are detected by listeners with and without language comprehension
Source: Commun Psychol. 2025 Apr 17;3:65. doi: 10.1038/s44271-025-00237-2 (PMC12006449; doi:10.1038/s44271-025-00237-2)
Supplement: Supplementary file 4 — Supplementary Table 1 [file 44271_2025_237_MOESM4_ESM.docx]

| Supplementary Table 1.  *Means (and Standard Deviations) of Vocal Characteristics for Accuracy and Confidence* | | | | | | |
| --- | --- | --- | --- | --- | --- | --- |
| Vocal characteristics | *Accuracy* | | | Witness Confidence | | |
| *Temporal cues* | Correct | Incorrect | *Cohen’s d* | 100 % confident | *< 100% confident* | *Cohen’s d* |
| VoicedSegPerSec | 1.83 (0.88) | 1.73 (0.88) | 0.11 | 1.85  (0.88) | 1.66  (0.84) | 0.22 |
| VoicedSegM | 0.29 (0.20) | 0.26 (0.18) | 0.16 | 0.31  (0.21) | 0.25  (0.15) | 0.33 |
| UnvoicedSegM | 0.36 (0.34) | 0.44 (0.46) | 0.20 | 0.32  (0.27) | 0.47  (0.46) | 0.40 |
| *Spectral balance cues* |  |  |  |  |  |  |
| F1 (amp) | -107.84 (41.84) | -121.18 (40.68) | 0.32 | -102.69 (41.88) | -125.17 (38.66) | 0.56 |
| AlphaRatio_UV | -16.06 (3.21) | -16.72 (3.02) | 0.21 | -15.82 (3.22) | -16.83  (3.09) | 0.32 |
| H1-H2 | 3.44 (5.01) | 3.99 (4.72) | 0.11 | 3.55  (4.71) | 3.69  (5.09) | 0.03 |
| MFCC3_V (M) | 9.49 (5.79) | 10.22 (6.44) | 0.12 | 9.11  (6.65) | 10.33  (5.65) | 0.20 |
| SpectralSlope_V | -0.02 (0.01) | -0.02 (0.01) | 0.00 | -0.02 (0.01) | -0.02  (0.01) | 0.00 |
| *Energy cues* |  |  |  |  |  |  |
| Int (M) | 0.38 (0.13) | 0.34 (0.12) | 0.32 | 0.39  (0.13) | 0.33  (0.11) | 0.50 |
| Int (SD) | 0.49 (0.14) | 0.48 (0.14) | 0.07 | 0.49  (0.13) | 0.49  (0.14) | 0.00 |
| *Frequency cues* |  |  |  |  |  |  |
| F0 (M) | 29.05 (5.00) | 29.05 (4.68) | 0.00 | 29.07 (4.80) | 29.35  (5.06) | 0.06 |
| F0 (SD) | 0.13 (0.07) | 0.13 (0.07) | 0.00 | 0.13  (0.07) | 0.14  (0.07) | 0.14 |
| F0 (rise) | 160.49 (168.16) | 178.49 (176.54) | 0.10 | 156.72 (169.87) | 174.28 (169.46) | 0.10 |
| F0 (fall) | 68.27 (105.34) | 69.70 (87.44) | 0.10 | 64.52 (100.04) | 76.62  (106.99) | 0.12 |
| F1 (M) | 470.69 (63.89) | 464.22 (56.60) | 0.11 | 473.13 (64.49) | 465.26  (59.16) | 0.13 |
| F3 (bw) | 826.19 (161.36) | 832.95 (144.27) | 0.04 | 834.98 (160.72) | 831.73 (152.22) | 0.02 |
